# Both Male and Female Malfunction Contributes to Yield Reduction under Water Stress during Meiosis in Bread Wheat

**DOI:** 10.3389/fpls.2016.02071

**Published:** 2017-01-10

**Authors:** Ifeyinwa Onyemaobi, Hui Liu, Kadambot H. M. Siddique, Guijun Yan

**Affiliations:** ^1^School of Plant Biology, Faculty of Science, The University of Western AustraliaCrawley, WA, Australia; ^2^The UWA Institute of Agriculture, The University of Western AustraliaCrawley, WA, Australia

**Keywords:** meiosis, seed set, *Triticum aestivum*, wheat, water stress, reproductive parts

## Abstract

Water stress during meiosis in wheat is a major constraint to yield especially for the rainfed farming regions. Pollen sterility has been proposed as the most sensitive process leading to low seed set (low % of fertile spikelets), but here we show this is not universal, and that the development of female reproductive parts is equally if not more sensitive than male parts in many wheat cultivars. The first experiment examined water stress during meiosis in 46 wheat genotypes. The reduction in seed set varied widely, ranging from 6 to 48%. The second experiment differentiated the effect of water stress on the male or the female reproductive part in 13 wheat genotypes. Water stress was imposed during meiosis, with plants cross-pollinated 5 days later with pollen from stressed or unstressed plants used to pollinate emasculated stressed or unstressed female parts. Seed set and kernel weight were measured at maturity. Contrary to the well-held view that the male reproductive part is the major contributor to seed set reduction when water stress is experienced during meiosis, the stressed-female part was also a predominant contributor in four wheat genotypes among the 13 genotypes examined. This strongly indicates that both male and female parts are responsible for yield reduction when water-stressed during meiosis and suggests that it may be possible to breed tolerant wheat cultivars combining tolerance from both male and female reproductive parts.

## Introduction

Water stress is a common abiotic stress limiting crop growth and productivity, and its duration and intensity are highly variable due to climate change. Improving yield is a major aim of most grain crop breeding projects. The extent of yield limitation due to water stress lays heavily on the particular growth stage at which plants experience the stress (Salter and Goode, 1967; Saini, 1997). Water stress during vegetative or reproductive phases can reduce yield (Saini and Westgate, 1999; Craufurd and Wheeler, 2009; Farooq et al., 2014), but the reproductive phase is the most critical stage (Parish et al., 2012). Water stress during or before the onset of reproductive process in plants is a major factor limiting crop yield in most regions where dryland rainfed farming occurs. Such regions experience water shortage and high temperatures as grain crops enter their reproductive stage (Siddique et al., 2000; Turner, 2003, 2004; Turner et al., 2007; Fang et al., 2010; Farooq et al., 2014).

The reproductive phase in bread wheat (*Triticum aestivum*) consists of a series of sequential events that span the Zadoks′ scale from Z37 (before booting) to Z69 (end of flowering) (Zadoks et al., 1974). This phase is sensitive to abiotic stresses and can result in yield losses in cereals (Oliver et al., 2005). A frequently adopted breeding approach for maximizing yield under water stress is to target a specific developmental stage prone to the stress and develop cultivars adapted to it (Fleury et al., 2010; Passioura, 2012). Grain number and grain weight are two major yield determinants in cereals (Evans, 1978; Mohammady, 2015). The timing of the water stress determines whether grain number (seed set) or grain weight will be affected. Water stress has the largest influence on seed set if it occurs during the earlier stages of the reproductive process (between meiosis and gametogenesis) (Saini and Westgate, 1999; Powell et al., 2012). Water stress during meiosis obstructs both male and female gametogenesis which may result in the production of sterile pollen and/or egg cells, loss of gamete viability, or other developmental anomalies of reproductive structures which reduce seed set (Saini, 1997; Saini and Westgate, 1999; Farooq et al., 2011, 2014).

Male gametophyte development in wheat is reportedly more sensitive to water, heat and cold stress than female gametophyte development (Saini et al., 1984; Dolferus et al., 2011). Water stress during the young microspore stage of pollen development in wheat can induce male sterility (Lalonde et al., 1997; Ji et al., 2010). The high sensitivity of male gametes to water stress has been attributed to the degradation of tapetum layers which contain the nutrients required for pollen development (Saini and Aspinall, 1981; Saini, 1997; Saini and Westgate, 1999; Dolferus et al., 2013). In contrast, female gametogenesis is reportedly physically protected, less vulnerable to abiotic stresses and resilient to water stress (Saini, 1997; Ji et al., 2010; Thakur et al., 2010; Dolferus et al., 2013).

This study was undertaken to examine how water stress during meiosis affects the male and female reproductive parts of wheat for their contributions to seed set and yield. The identification of wheat genotypes with high seed set under water stress during meiosis could be the key to further improving drought tolerance in wheat. The specific objectives of the research were to: (i) identify wheat genotypes with high seed set under water stress during meiosis, (ii) determine any genotypic differences in floral fertility under meiotic-stage water stress, and (iii) investigate the contribution of male and female reproductive parts on seed set and yield in response to water stress during micro- and mega-sporogenesis.

## Materials and Methods

### Experiment 1: Effect of Water Stress during Meiosis on Seed Set

#### Plant Materials and Growing Conditions

Forty-six wheat (*Triticum aestivum* L.) genotypes were grown in a controlled-temperature glasshouse, set at 22/15°C day/night temperature at The University of Western Australia (31° 57′ S, 115° 47′ E) from June to November 2014 (**Table 1**). The seeds were obtained from the Australian winter cereals collection.

**Table 1:** Names and origins of the 46 wheat genotypes used in this study.

S/N	Name	Country/Continent	Habitat	Days to physiological maturity		S/N	Name	Country/Continent	Habitat	Days to physiological maturity	
				Control	Water stressed					Control	Water stressed
1	Abura^∗^	Japan-Asia	Spring	117	119	24	India 344	India-Asia	Spring	111	114
2	AUS 12351^∗^	Argentina-South America	Spring	113	111	25	Israel L224	Israel-Middle East	Spring	119	116
3	AUS 12671^∗^	Mexico-North America	Winter	117	117	26	Janz	Australia-Australia	Spring	116	111
4	Axe	Australia-Australia	Spring	111	111	27	Jimai 20^∗^	China-Asia	Spring	119	125
5	Beijing 8	China-Asia	Winter	157	157	28	Kenya 1877^∗^	Kenya-Africa	Spring	109	109
6	Belgrade 7	Yugoslavia-Europe	Winter	144	144	29	Kenya Bongo	Kenya-Africa	Spring	187	185
7	Canary 4	Spain-Europe	Spring	177	177	30	Kukri	Australia-Australia	Spring	109	111
8	Changli^∗^	China-Asia	Spring	102	98	31	Magenta	Australia-Australia	Spring	118	118
9	Diamante inta	Argentina-South America	Spring	105	105	32	Morocco 426^∗^	Morocco-Africa	Spring	142	134
10	Emu Rock^∗^	Australia-Australia	Spring	98	97	33	Nobre	Brazil-North America	Spring	120	120
11	Erechim^∗^	Brazil-South America	Spring	131	130	34	Norin 10	Japan-Asia	Winter	122	122
12	Espada	Australia-Australia	Spring	114	114	35	Persia 123	Iran-Middle East	Spring	161	161
13	Fang 60	Thailand-Asia	Spring	109	107	36	Philippines 7	Philippines-Asia	Spring	120	120
14	Flaminio	Italy-Europe	Spring	137	118	37	Punjab 8A^∗^	India-Asia	Spring	125	125
15	Florida 301	USA-North America	Winter	134	127	38	Riddley	India-Asia	Spring	131	131
16	Funello	Italy-Europe	Winter	113	107	39	RL 6019^∗^	Canada-North America	Spring	139	131
17	Gail	South Africa-Africa	Winter	97	97	40	Sakha 8	Egypt-Africa	Spring	102	102
18	Galaxy H45	Australia-Australia	Spring	113	109	41	Spoetnik^∗^	South Africa-Africa	Spring	113	116
19	GBA Sapphire	Australia-Australia	Spring	106	107	42	Tammarin Rock	Australia-Australia	Spring	105	108
20	Gilat 182	Israel-Middle East	Spring	97	97	43	Thatcher	USA-North America	Spring	156	152
21	Giza 150	Egypt-Africa	Spring	116	102	44	W96	Pakistan-Asia	Spring	152	147
22	Halberd	Australia-Australia	Spring	140	134	45	Westonia^∗^	Australia-Australia	Spring	109	112
23	Hybride 38	India-Asia	Spring	114	108	46	Yaqui 50	Mexico-North America	Spring	106	103

*^∗^Genotypes also used in Experiment 2*.

Plants were grown in polyvinyl chloride pots, 9 cm in diameter and 37 cm high. Each pot was filled with 2.1 kg of sterilized potting mix and had a 9-mm hole at the bottom to allow free drainage of water. Five seeds were grown per pot for each genotype and thinned to three uniform plants per pot after 7 days. There were 276 pots in total; two treatments × 46 genotypes × 3 replicates. To maintain soil moisture and avoid quick soil drying, 100 g of plastic poly pellets was added on the soil surface of each pot. A water-soluble NPK fertilizer (poly feed greenhouse grade) was applied weekly from 21 days after sowing (DAS).

#### Water-Stress Treatments

For each genotype, three pots were grown as replicates in each treatment: non-stress (control) or stress. The pots were watered every 2 days to maintain soil moisture at 80% field capacity. The pots were randomly arranged and rearranged every fortnight to reduce border effects and minimize any variation in light and temperature, as described in Fang et al. (2010).

**FIGURE 1:**
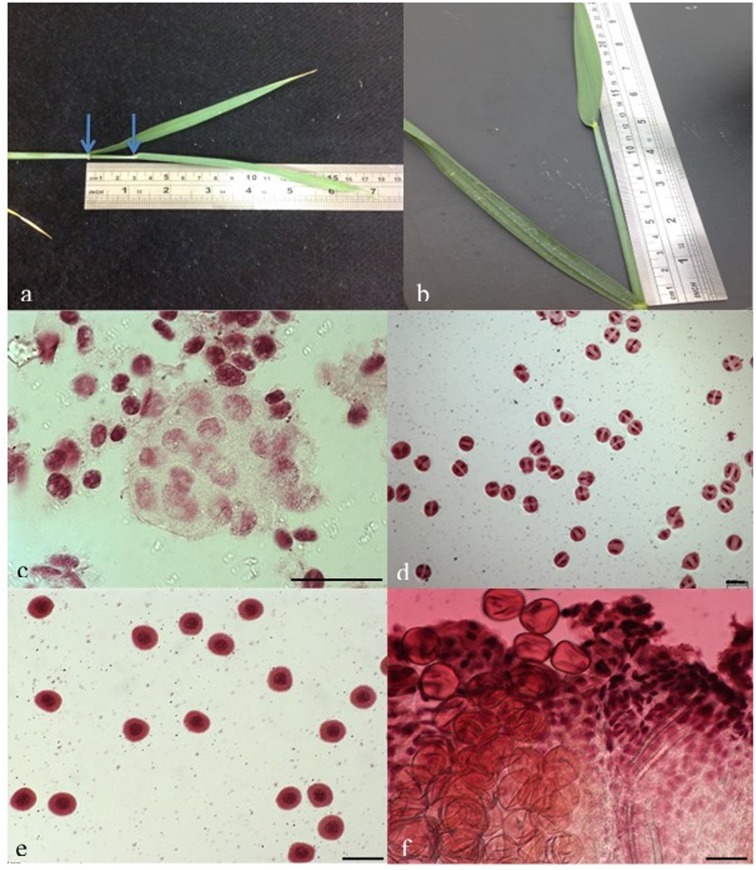
**Correlation of auricle distance (AD) to meiosis stage in wheat. (a)** AD = ∼3 cm when meiosis started for most genotypes; **(b)** AD = ∼12 cm when meiosis ended for most genotypes; **(c)** Microscopic examination of anthers at AD < 3 cm showing pollen mother cells (the lightly stained large cells in the middle) before meiosis; **(d,e)** Microscopic examinations of anthers at AD between 3 and 12 cm, showing cells **(d)** at different meiotic stages and **(e)** having just finished meiosis; **(f)** Microscopic examination of anthers at AD ≥ 12 cm, showing pollens that had already formed. Bars = 50 μm.

Water stress was imposed in the stress group by the cessation of watering for 7 days when the auricle distance (AD, the distance between the auricles of the flag leaf and the second last leaf, as described by Ji et al., 2010) was 0 cm. At this time (day 7 after stress imposition), the AD was ∼12–14 cm (**Figures 1a,b**) when meiosis occurred (**Figures 1c–f**) for most genotypes. Watering was then resumed as per the control pots and continued until the plants reached full maturity.

### Experiment 2: Effect of Water Stress on the Reproductive Parts of Selected Genotypes

**FIGURE 2:**
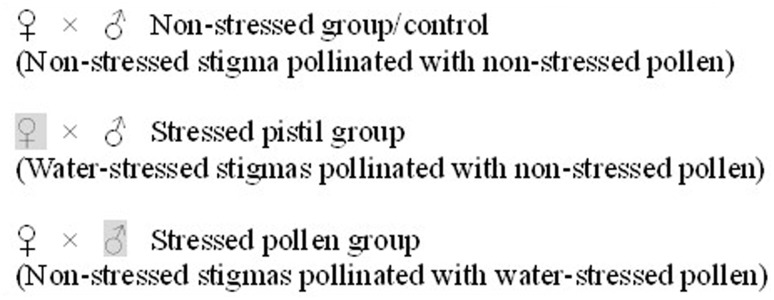
**List of treatment groups and crosses performed**. Water-stressed female and male parts are shaded in gray.

To determine the effect of water stress on the reproductive parts of wheat, 13 genotypes (**Table 1**) were selected from Experiment 1 based on the following criteria: high seed set in the non-stressed treatment (control), and extremely high or low reductions in seed set in the stressed treatment relative to the control. A similar water-stress treatment to Experiment 1 was used, but this time the non-stressed and water-stressed plants were reciprocally crossed by hand for each of the selected genotypes. Spikelets at the top and base of the ear, and florets from the central part of the spike were removed. A total of 20 spikelets from the middle of the spike and 10 lateral spikelets on each side of the spike were retained. Emasculation was done on all the 20 spikelets on the main stem only. Three treatment groups were set up: pistils/stigmas of non-stressed plants were pollinated with pollen from non-stressed plants, as a control group; stigmas of water-stressed plants were pollinated with non-stressed pollen, as a stressed-female (pistil) group; and stigmas from non-stressed plants were pollinated with pollen from water-stressed plants, as a stressed-male (pollen) group (**Figure 2**). For each genotype, three replicates were used for each of the treatment groups.

#### Measurements and Statistical Analysis

Each main stem was tagged at the beginning of the water-stress treatment, and the effect of the stress was assessed for the tagged stem only. Soil water content (SWC) was measured to determine plant water-stress status during the stress treatment. SWC was determined by oven-drying a soil sample to constant weight at 105°C and the moisture content of the soil was expressed as a percentage of the sample weight before and after drying. For both treatments, harvest occurred at the end of physiological maturity, when the spikes were dry and with a golden color. For each genotype and treatment, the following parameters were measured in three plants and three replicates:

$$\text{Seed set}=\frac{\text{Number of fertile florets that developed into grains}}{\text{Number of potentially fertile florets}}$$

$$\text{Seed set reduction index}=\;\frac{\left(\text{seed set of control plants}-\text{seed set of stressed plants}\right)}{\text{seed set of control plants}}$$

The number of potential fertile florets was determined by counting the flowers per spike after anthesis and at the end of physiological maturity.

Thousand kernel weight, plant height, ear dry weight, flag leaf length, flag leaf width, the number of nodes per plant and peduncle length were also measured. Statistical analyses were performed using SPSS 22.0. Analysis of the interaction effect on the measured traits because of the water-stress treatment, analysis of variance and *t*-test were conducted. Differences between mean values of treatments were evaluated using least significant difference (LSD) at the 0.05 significance level. Pearson’s correlation coefficient was used to identify relationships between the measured characters.

## Results

### Water Stress Significantly Reduced Seed Set

**FIGURE 3:**
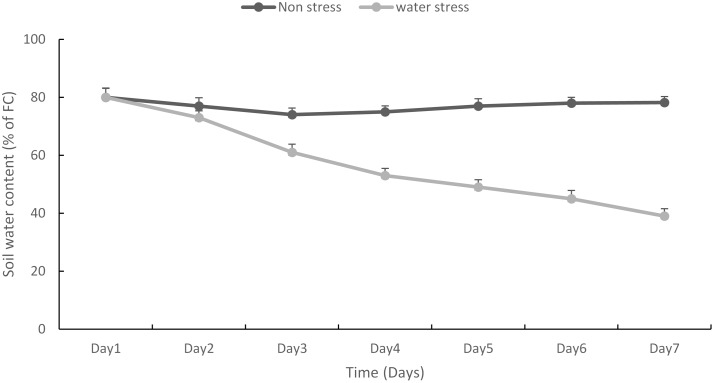
**Change in the soil water content (SWC) [% of field capacity (FC)] with time (days) for non-stressed (upper line) and water stressed (lower line) treatment after watering was withheld for 7 days**. Values are means; error bars are standard errors of the means.

Cessation of watering rapidly decreased SWC from 80% field capacity to 61% in 3 days and 45% in 7 days (**Figure 3**). Water stress during meiosis significantly reduced (*p* < 0.01) seed set compared to non-stressed plants. For the 46 tested wheat genotypes, the mean seed set under non-stressed conditions was 68%, because not all fertile florets had the potential of setting into grains, similar result has been previously reported in Ferrante et al. (2013) where increase in nitrogen availability affected the number of fertile florets and the number of grains the florets produce at maturity - the relationship of wheat spike fertility and seed set was also reported by Martino et al. (2015). Under stressed conditions the mean seed set was reduced to 52%.

**FIGURE 4:**
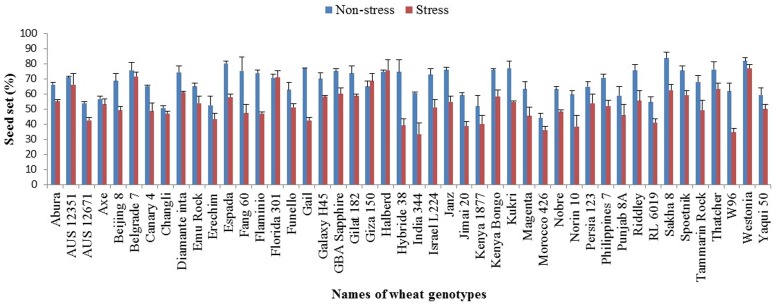
**Seed set in 46 wheat genotypes under water-stressed and non-stressed (control) conditions**. Values are means; error bars are standard errors of the means.

Lower seed set values (ranging from 33 to 77%) were recorded under water-stressed conditions for the 46 wheat genotypes (**Figure 4**). Florida 301, Halberd and Westonia had the highest seed set with >70% under water stress. These values did not significantly differ from those under control conditions. India 344, Morocco 426 and W96 had the lowest seed set which ranged from 33 to 40% under water stress.

Under non-stressed conditions, 25 of the 46 wheat genotypes had a mean seed set >70%. Westonia, Sakha 8 and Espada had seed sets of >80% while Morocco 426 had the lowest seed set of <50%.

For some genotypes, no significant difference in seed set was observed between the water stressed and control treatment (**Figure 4**), suggesting water-stress tolerance mechanism might exist in these genotypes which can be used for further studies. The mean seed set per spike under water stress during meiosis was reduced by 24% compared with the well-watered control.

### Water Stress during Meiosis and Seed Weight

The mean 1000 kernel weight (TKW) of the 46 genotypes did not significantly differ (*p* < 0.01) between the stressed and control treatments. Under water stress, the mean TKW was 23.69 ± 3.61 g while the control treatment was 24.27 ± 3.70 g. Emu Rock and Spoetnik had the highest TKWs under control conditions (48.6 and 41.3 g, respectively). Apart from a few genotypes, such as Thatcher and RL 6019 which had the lowest TKWs (3.24 and 1.90 g, respectively) when stressed, the TKW of most of the tested genotypes did not significantly differ between the two treatments.

### Water Stress and the Development of Both Male and Female Parts

**FIGURE 5:**
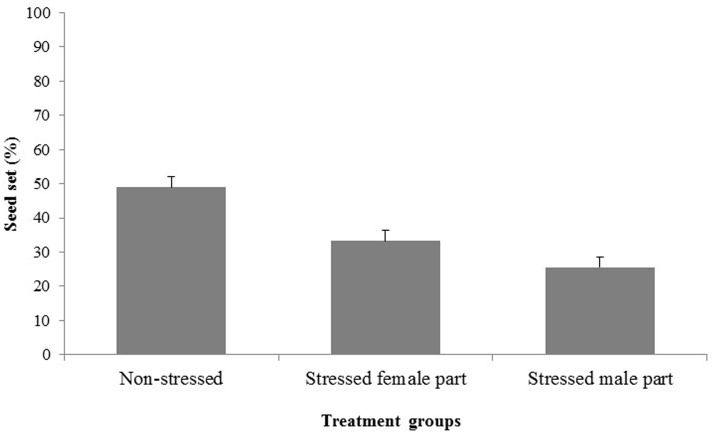
**Seed set (%) in 13 wheat genotypes screened under treatments of non-stressed (control), water-stressed female parts (pistil) and water-stressed male parts (pollen)**. Values shown are means; error bars are standard errors of the means. ANOVA indicated significant differences at the *p* < 0.01 level for all treatment groups.

In Experiment 2, seed set differed significantly (*p* < 0.01) when the male and female parts were independently stressed compared with that of the control group (**Figure 5**). The stressed-female group recorded a mean seed set of 33% while the stressed-male group recorded 26%; which reflected a respective 48 and 32% reduction in seed set compared with the control group.

**FIGURE 6:**
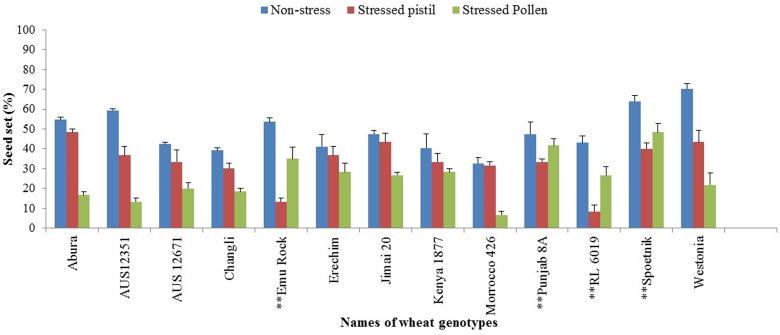
**Seed set performance in selected wheat genotypes under different treatment conditions**. Values shown are means, and error bars are standard errors of the means. ^∗∗^Genotypes whose water-stressed female reproductive part predominantly contributed to low seed set while those without asterisk are genotypes whose water-stressed male reproductive part was the major contributor to low seed set.

Four of the 13 selected wheat genotypes—Emu Rock, Punjab 8A, RL 6019 and Spoetnik—had lower seed sets under the stressed pistil (female) treatment compared with their stressed pollen (male) counterparts (**Figure 6**). Even when these genotypes were pollinated with non-stressed pollen in the stressed pistil treatment, water stress had a significant effect (*p* < 0.01) on seed set as it reduced the number of seeds per spike. For the other nine genotypes, the stressed pollen treatment recorded significantly (*p* < 0.01) lower seed set than the stressed pistil treatment (**Figure 6**).

Compared with the seed set recorded under control conditions, some genotypes had a high reduction in seed set under one treatment condition but not in the reciprocal treatment. For example, in the stressed-female treatment, RL 6019 had a seed set reduction of 81%, but only 38% was recorded in the stressed-male treatment. Similarly, but in reverse, Morocco 426 recorded an 80% reduction in seed set in the stressed-male treatment and a non-significant 3% reduction in the stressed-female treatment.

### Other Traits Affected by Water Stress during Meiosis

Physiological traits that could affect seed set were measured to identify their relationship with seed set under the imposed stress condition, including plant height, number of nodes per plant, and flag leaf dimensions (length and width) which could influence AD measurement as it was based on the growth of the flag leaf. Water stress during meiosis significantly (*p* < 0.01) affected the mean values between the stressed and non-stressed treatments for ear dry weight, flag leaf width, flag leaf length, peduncle length, and plant height in the 46 genotypes screened.

In Experiment 2, the correlation analysis of the measured traits showed that ear dry weight was positively correlated with seed set for both stressed-male and stressed-female treatments. Significant correlations were observed under the stressed pollen (male) treatment (*p* < 0.05, *r* = 0.31) and stressed pistil (female) treatment (*p* < 0.01, *r* = 0.58) (**Table 2**). Peduncle length had a significant negative association (*r* = -0.39) with seed set in the non-stressed treatment, but a significant positive correlation (*r* = 0.44) in the stressed-female treatment and no association in the stressed-male treatment. The number of nodes had no significant correlation with seed set for all treatments as predicted, as it was determined prior to reproductive stage. Plant height had a significant negative correlation (*p* < 0.01, *r* = –0.34) with seed set in the non-stressed treatment, but no significant relationship was observed under stressed conditions.

**Table 2:** Correlation of seed set (SES), ear dry weight (EDW), flag leaf width (FLW), plant height (PHT), number of nodes per plant (NON), peduncle length (PDL) and flag leaf length (FLL) under non-stressed, water-stressed female (pistil) and water-stressed male (pollen) treatments.

	SES	EDW	FLW	PHT	NON	PDL	FLL	
**Non-stress**								
SES	1							
EDW	0.140	1						
FLW	0.275^∗^	0.348^∗∗^	1					
PHT	-0.337^∗∗^	0.030	-0.147	1				
NON	-0.165	-0.268^∗^	-0.146	0.681^∗∗^	1			
PDL	-0.390^∗∗^	0.468^∗∗^	0.240	0.222	-0.273^∗^	1		
FLL	-0.026	0.248^∗^	0.319^∗^	-0.082	-0.173	0.325^∗∗^	1	
**Stressed pistil**								
SES	1							
EDW	0.584^∗∗^	1						
FLW	-0.259^∗^	-0.001	1					
PHT	0.204	0.179	-0.019	1				
NON	0.007	0.144	0.005	0.678^∗∗^	1			
PDL	0.435^∗∗^	0.110	0.182	-0.180	0.214^∗^	1		
FLL	-0.311^∗^	-0.150	0.204	-0.100	-0.276^∗^	0.266^∗^	1	
**Stressed pollen**								
SES	1							
EDW	0.311^∗^	1						
FLW	-0.105	0.050	1					
PHT	0.049	-0.136	0.131	1				
NON	-0.043	-0.227	0.163	0.673^∗∗^	1			
PDL	0.071	-0.059	-0.089	-0.006	-0.206	1		
FLL	-0.246	-0.055	0.262^∗^	-0.140	-0.227^∗^	0.071	1	

*^∗^Correlation significant at the 0.05 level (two-tailed); ^∗∗^correlation significant at the 0.01 level (two-tailed)*.

## Discussion

The observed differences in seed set in the non-stressed and stressed treatments in this study highlighted the high sensitivity of the meiotic process to water stress. Even though normal watering resumed after 7 days of water withholding, the stress during meiosis had a significant and irreversible effect on seed set. This result was consistent with previous reports on water stress during meiosis from Ji et al. (2010), Thakur et al. (2010), and Dolferus et al. (2013).

Male gametophyte development in wheat is reportedly more sensitive to water stress than the female reproductive part which is considered to be resilient to water stress during meiosis because the ovule is inverted, the micropyle bent down to the funiculus to which the body of the ovule is joined (termed as “anatropous” type of ovule) (Ji et al., 2010; Thakur et al., 2010; Dolferus et al., 2013). Pollen sterility is regarded as the major contributor to poor grain set in water-stressed wheat crops (Saini et al., 1984; Lalonde et al., 1997; Ji et al., 2010; Dolferus et al., 2011). Our series of newly designed reciprocal crosses using stressed genotypes to cross with their non-stressed “selves,” allowed the comparison of stressed-male only or stressed-female only treatments with the non-stressed treatment. Seed set reduction index was used to identify genotypes with high/low seed set rates under water stress. We found that both male and female parts can contribute to seed set reduction under water stress during meiosis. Although, most of the tested genotypes (9 of 13) demonstrated more sensitivity in male parts than female parts, four genotypes—Emu Rock, Punjab 8A, RL 6019 and Spoetnik—showed more sensitivity in female parts than male parts (**Figure 6**). When pollinated with non-stressed viable pollen, these highly sensitive female parts resulted in a low seed set. This is a clear indicator of the different sensitivities of male and female parts to water stress during meiosis in those wheat genotypes.

A plant’s response to water stress can be at the cellular, physiological, or molecular levels (Barnabas et al., 2008). Examples of such responses during meiosis include the reduced competitiveness of different floral organs to attract and store nutrients, inhibition of photosynthetic processes which reduces nutrient supply to reproductive organs, and accumulation of high abscisic acid (ABA) concentration (Ji et al., 2010; Alqudah et al., 2011). Depending on the duration and intensity of the stress, plants change the way their genes express and produce certain enzymes or proteins that are specific to the tissues and the prevailing stress condition, which could affect the performance of either or both the male and female reproductive parts. The determination of resilience/vulnerability of the reproductive parts in previous reports—based either on physical structures or outcrossing abilities—may not reflect the actual cellular status under the stress during meiosis. Also, due to the difficulty in assessing the viability of female reproductive parts, the reports based on outcrossing ability could lead to a biased conclusion. We suggest that there might be physical variations of the reproductive organs among genotypes. In some genotypes, at least, female part is more vulnerable than male part to the stress that causes unrecoverable meiosis damage. As the female part can only produce a limited number of eggs, in contrast to the large number of pollen that the male part can produce, it can become a decisive factor for plant performance under stress.

**FIGURE 7:**
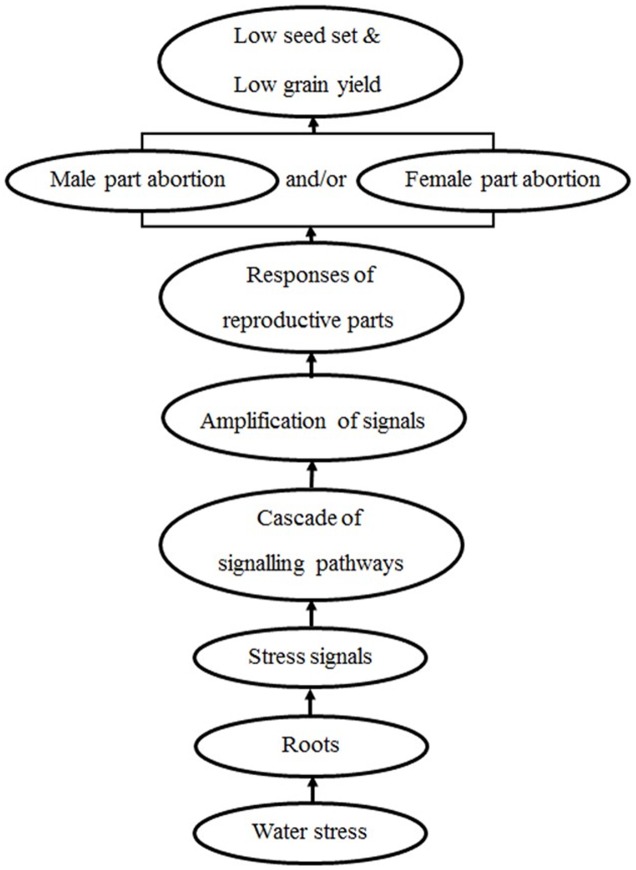
**Proposed model for signal induction and transduction in response to meiotic-stage water stress in wheat**. Water stress is sensed by roots and signals are sent to the above ground resulting in the abortion of male and/or female parts and low seed set/yield.

It was reported that male and female meiosis occurs at about the same time under normal conditions, but there is asynchrony under stress conditions (Bennett et al., 1973). The differential sensitivity of the male and female organs observed could also be caused by a shift in organ development and timing of meiosis resulting in altered water stress sensitivity, which suggests that there are factors other than physical structures affecting the developmental processes of reproductive parts. Our results indicate that both male and female parts could be sensitive to water stress during meiosis, and either male, female, or both parts could be the major contributor to yield reduction in different wheat genotypes. This suggests that different cellular responses to the stress signal rather than different physical structures between male and female reproductive parts are the main reason for the different performances of the two parts under water stress. We propose a simple model for signal initiation and transduction in response to meiosis-stage water stress in wheat (**Figure 7**). We hypothesize that when water stress coincides with meiosis in wheat plants, stress signals are sent from the roots when the available water content falls below a critical threshold level, which initiates a cascade of specific signal transduction pathways which in turn change the cellular signals. To ensure survival and/or tolerance, the different signaling molecules upregulated by water stress cause the plant to respond by aborting either the male, female or both reproductive organs to ensure that some seeds can grow for the next generation but results in low seed set and ultimately low grain yield. Although, the duration of the water stress was only for 7 days, the level of the stress was sufficient to make pollen and ovary incompetent; if the stress coincides with meiosis (the total meiotic duration in common wheat is approximately 24 h) when male and female parts are at the most sensitive development stage. Based on this model, the regulated signal transduction of the reproductive parts under stress may be responsible for the observed variation in seed set performance of different genotypes. As different wheat genotypes have different sets of genes which follow different signaling pathways, the male and female reproductive parts in each genotype may perform differently in how they perceive and transmit water stress signals during meiosis to influence the final seed set.

Based on the results of this study, we can also conclude that selective screening of seed set for wheat lines that are tolerant to water stress during meiosis is a promising way to breed drought-tolerant lines. Water stress during meiosis reportedly affects seed set rather than seed size (Alqudah et al., 2011; Guo and Schnurbusch, 2015). Not surprisingly, this study found no significant differences in seed size between the stressed and non-stressed treatments for most of the tested genotypes. Seed set (grain number) is a determining component of grain yield in wheat in rainfed areas. Australian dryland wheat yields about 2–2.5 t ha^-1^ while irrigated wheat can reach 10 t ha^-1^, but there is little difference in quality (seed size) (ABARES, 2014), suggesting that the lower yield in dryland wheat is mainly due to reduced seed numbers, not seed size.

In summary, this study showed that high genotypic variation exists in different wheat genotypes for seed set performance under water stress during meiosis. We identified, for the first time, that the stressed-female reproductive part was one of the major contributors to low seed set in wheat genotypes water-stressed during meiosis. To further extend this research, wheat genotypes with extremely sensitive/resilient female or male parts to water stress during meiosis could serve as useful germplasm for analyzing the underlying genetic mechanisms. The genotypes with the most contrasting seed set data from this study are being crossed to obtain segregating populations for drought tolerance breeding and future genetic studies.

## Author Contributions

GY and KS conceived and designed the experiments. IO conducted the major experiments and HL conducted part of the cytogenetic study. IO and HL analyzed the data and wrote the manuscript. GY and KS critically reviewed the manuscript. All authors approved the final version of the manuscript.

## Conflict of Interest Statement

The authors declare that the research was conducted in the absence of any commercial or financial relationships that could be construed as a potential conflict of interest. The reviewer JR and handling Editor declared their shared affiliation, and the handling Editor states that the process nevertheless met the standards of a fair and objective review. The reviewer RM declared a shared affiliation, though no other collaboration, with the authors to the handling Editor, who ensured that the process nevertheless met the standards of a fair and objective review.
